# Emotional Status in Relation to Metacognitive Self-Awareness and Level of Functional Disability Following Acquired Brain Injury

**DOI:** 10.3390/brainsci15080841

**Published:** 2025-08-06

**Authors:** Valentina Bandiera, Dolores Villalobos, Alberto Costa, Gaia Galluzzi, Alessia Quinzi, Arianna D’Aprile, Umberto Bivona

**Affiliations:** 1 Department of Human Science, LUMSA University, 00193 Rome, Italy; v.bandiera1@lumsa.it; 2 Department of Experimental Psychology, School of Psychology, Complutense University of Madrid, 28223 Madrid, Spain; marvil17@ucm.es; 3 Center for Cognitive and Computational Neuroscience, Complutense University of Madrid, 28015 Madrid, Spain; 4 Institute of Knowledge Technology, Complutense University of Madrid, 28223 Madrid, Spain; 5 Department of Economic, Psychological, Communication, Educational and Motor Sciences, Niccolò Cusano University, 00166 Rome, Italy; alberto.costa@unicusano.it; 6 Department of Psychology, “Sapienza” University of Rome, 00185 Rome, Italy; 7 IRCCS Istituto delle Scienze Neurologiche di Bologna, 40139 Bologna, Italy; 8 Independent Researcher, 00100 Rome, Italy; aridap1999@gmail.com; 9 IRCCS Fondazione Santa Lucia, 00179 Rome, Italy

**Keywords:** moderate-to-severe acquired brain injury, self-awareness, anosognosia, anosodiaphoria, emotional reactions, functional disability, post-traumatic stress disorder

## Abstract

**Background/Objectives**: Impairment in self-awareness (ISA) is one of the common consequences of an acquired brain injury (ABI) and is associated with anosodiaphoria. Collectively, these co-occurring neuropsychological disorders pose significant obstacles in the neurorehabilitation of moderate-to-severe ABI patients. Individuals who recover from ISA may present with anxiety and/or depression as adaptive reactions to the ABI, along with related functional disabilities. The present study investigated whether the level of metacognitive self-awareness (SA) is associated with the presence of anxiety and depression, apathy, or anosodiaphoria in patients with moderate-to-severe ABI. It aimed also at investigating the possible relationship between the severity of disability and both psycho-emotional diseases and the presence of PTSD symptoms in patients with high metacognitive SA. **Methods**: Sixty patients with moderate-to-severe ABI and different levels of metacognitive SA completed a series of questionnaires, which assessed their self-reported metacognitive SA, anosodiaphoria, anxiety and depression, apathy, and PTSD symptoms. **Results**: Low-metacognitive-SA patients showed lower levels of anxiety and depression and higher anosodiaphoria than high-metacognitive-SA patients. Patients with high metacognitive SA and high levels of disability showed significant higher states of anxiety and PTSD symptoms than patients with high metacognitive SA and low levels of disability. **Conclusions**: The neurorehabilitation of individuals with moderate to severe ABI should address, in particular, the complex interaction between ISA and anxiety and depression in patients during the rehabilitation process.

## 1. Introduction

One of the more frequent consequences of a moderate-to-severe acquired brain injury (ABI) is the impairment of self-awareness (SA) [1,2]. Prigatano and Schacter [3] defined SA as the “capacity to perceive the ‘self’ in relatively ‘objective’ terms while maintaining a sense of subjectivity”.

The impairment of self-awareness (ISA) after ABI involves many different functions, e.g., cognitive, motor, social judgment, behavioural, and overall level of functional competency in everyday life [1,4,5,6,7]. This may cause failure to adopt adequate compensatory strategies, and even the implementationof ineffective or dangerous behaviours, which negatively affect the patient’s functional outcome [8,9,10]. ISA after moderate-to-severe ABI may continue after patients have been discharged home [11]; this can remain for more than 5 years [12], or be lifelong [13].

Some theoretical models have attempted to describe SA. Among them, Crosson et al. [14] proposed a pyramidal system. The base of the pyramid is *intellectual* SA—the patient’s ability to understand that a particular function is impaired, as well as their ability to understand the functional implications of one’s deficits. The second level, *emergent* SA, is the patient’s ability to recognize a problem when it occurs. The highest level of the Crosson et al. model is *anticipatory* SA—the patient’s ability to (a) anticipate that a problem will occur as the result of some deficit, (b) realize in advance of their actions that a given deficit will cause a particular problem in the future, and (c) realize beforehand that a given compensation would reduce the chances of a problem occurring [14].

Another model, the Dynamic Comprehensive Model of Awareness (DCMA) [15], is a non-hierarchical model of SA. The DCMA conceptualizes *metacognitive* awareness—knowledge of task characteristics and knowledge of one’s own capabilities (similar to the concept of intellectual SA in the Crosson et al. model) and *online self-monitoring*—the patient’s ability to judge their own capabilities and limitations (i.e., during a performance) in relation to the current situation. Patients with good self-monitoring skills are able to detect their errors (similarly to the aforementioned concept of emergent SA), as well as appraise the demands of the current task (similarly to the aforementioned concept of anticipatory SA). Compared to the Crosson et al. [14] model, Toglia and Kirk view the relationship between different aspects of metacognition and SA as a dynamic process rather than as a series of hierarchical levels.

These models, however, do not address the repeated clinical observation that ABI patients often demonstrate some change in their emotional status or mood [16,17]. Although ISA is one of the main challenges faced in neurorehabilitation, it is often underestimated [2,10,18], since, in many cases, the rehabilitation protocols aim at treating the specific deficits in each functional domain (e.g., cognitive, emotional–behavioural, neuromotor, etc.), regardless of the specific patient’s SA level for one or more of such deficits [19].

Different emotional features have been reported in relation to different levels of SA, regardless of the ABI severity. An increase in SA is often associated with an increase in self-reported emotional distress [20]. High SA after ABI has been associated with depression [17,21,22,23,24], anxiety [17,24,25,26], and lower self-esteem [22,24,27].

Bivona et al. [17] also reported that patients with ISA tended to be more apathetic and alexithymic than those with high SA. Importantly, in their study, patients with ISA showed significantly higher scores in self-reported anosodiaphoria [17], a phenomenon first noted by Babinski in 1914 and characterized by affective indifference towards brain injury outcomes [28]. These two interrelated disorders pose major motivational barriers to successful rehabilitation [23,25,29,30]. Indeed, a recent study [18] conducted on patients with severe ABI and healthy controls showed that, compared to both high-SA-patients and healthy controls, low-SA-patients demonstrated not only greater anosodiaphoria, but also a lower level of functioning in everyday life.

In high-SA-patients with moderate-to-severe ABI, the influence of functional disability on the psycho-emotional state has not been thoroughly investigated. Fann et al. [31] and Malec et al. [32] found that higher levels of functional disability were associated with higher levels of anxiety and depression. However, these studies were conducted on patients with varying degrees of ABI severity and assessed functional disability exclusively through subjective measures of post-injury sequelae.

Similarly, the role of functional disability on the subjective experience of post-traumatic stress disorder (PTSD) symptoms requires further investigation. Villalobos and Bivona, [33] documented that PTSD symptoms can occur in patients who suffered and recovered from post-traumatic amnesia (PTA). However, no studies to date have investigated the relationship between PTSD symptoms and the level of functional disability in high-SA-patients.

In the present study, we evaluated whether the level of intellectual/metacognitive (“metacognitive” from now on) SA was associated with different levels of anxiety and depression, and/or anosodiaphoria and apathy. We predicted that, when compared to patients with higher levels of metacognitive SA, low-metacognitive-SA patients would show lower levels of anxiety and depression, and higher levels of apathy or anosodiaphoria.

The study also explored the interaction between level of functional disability and some emotional correlations in patients with high metacognitive SA. We predicted that the higher the level of functional disability, the more present the anxiety and depression. We also predicted that the higher the level of functional disability, the higher the subjective traumatic impact of the event (i.e., the greater the PTSD symptoms).

## 2. Materials and Methods

### 2.1. Participants

#### 2.1.1. Patients

Sixty-four patients with moderate-to-severe ABI due to different aetiologies [traumatic brain injury (TBI; n = 20); haemorrhage (n = 16); ischemia (n = 23), and cerebral tumour (n = 1)] who had been consecutively admitted to the Post-Coma Unit of Santa Lucia Foundation in Rome (Italy) from June 2022 to October 2024.

After enrolment, we excluded four patients from the study for the following reasons: lack of informal caregiver (“caregiver” from now on) availability (n = 2), and patient fatigue (n = 2). Thus, the final sample consisted of 60 patients (40 males and 20 females), with a mean age of 42.62 years (SD = 15.89) (ranging from 18 to 65 years), a mean educational level of 13.1 years (SD = 2.89) and a mean chronicity of 169 days (SD = 163.4). In total, 41 were inpatients and the remaining 19 were undergoing neurorehabilitation treatment in a day hospital (DH) program.

Participants were recruited according to the following inclusion criteria: (1) age ≥ 18 years; (2) diagnosis of moderate-to-severe ABI [34] score between 9 and 12 (moderate ABI) or ≤8 (severe ABI) in the acute phase]; (3) Levels of Cognitive Functioning (LCF) scale [35] score ≥ 7; (4) PTA resolution, when present in the post-acute phase; (5) distance from the ABI of at least one month; (6) ability to undergo formal psychometric evaluation despite cognitive and sensory–motor deficits; (7) availability of informed consent.

Exclusion criteria for patients were as follows: (1) a history of drug and alcohol addiction; (2) psychiatric diseases; (3) severe aphasia; (4) a history of neurological disorders; (5) recent psychological traumas (e.g., bereavement).

#### 2.1.2. Caregivers

To evaluate patients’ level of metacognitive SA, according to the discrepancy between their report and that of the caregivers on the Patient Competency Rating Scale -Neurorehabilitation Form (PCRS-NR) [36], 60 first-degree relatives (aged ≥ 18 years) were enrolled: 22 (36.7%) were parents of the patients, 23 (38.3%) were partners, 9 (15%) were children, and 6 (10%) were siblings. All relatives were caregivers of their loved one (i.e., they continuously assisted them in the hospital or at home when they were treated in DH), and accordingly they knew the patient very well. All caregivers gave their informed consent to participate in the study.

The study was approved by the Ethics Committee of Santa Lucia Foundation (Project identification code: CE/Prog.880; approval date: 28 November 2022). All participants signed a consent form for participation.

### 2.2. Procedure

After the enrolment, each participant was informed regarding all the characteristics of the study and, accordingly, informed consent was obtained by participants.

To administer the PCRS-NR, relative form, a psychologist met each care giver once, in a quiet room. Another psychologist met each patient once or twice, according to their degree of fatigue, to administer the PCRS-NR, patient form, and other questionnaires investigating the levels of anxiety, depression, apathy, and PTSD symptoms.

The level of metacognitive SA was determined by the discrepancy between the PCRS-NR relative form and patient form (PCRS-NR^DS^). A cut-off = 5 [18,37] was established to divide the sample into two groups: 43 high-metacognitive-SA patients (i.e., patients with a PCRS-NR^DS^ under the cut-off) and 17 with low metacognitive SA (i.e., patients with a PCRS-NR^DS^ over the cut-off) (Group 1).

High-metacognitive-SA patients were further divided into two subgroups according to their level of functional disability, based on the Disability Rating Scale (DRS) score [38] (see below for the description of the scale): Group 2, composed of 15 patients with a DRS score ranging from 7 to 21 (moderately severe-to-severe disability), and Group 3, composed of 28 patients with DRS score ranging from 0 to 6 (none-to-moderate disability).

The final groups, based on the PCRS-NR and DRS scores, were as follows:

- Group 1: *low*-metacognitive-SA patients (N = 17);
- Group 2: *high*-metacognitive-SA patients and *moderately severe-to-severe* disability (N = 15);
- Group 3: *high*-metacognitive-SA patients and *none-to-moderate* disability (N = 28).

### 2.3. Measures

#### 2.3.1. Functional Assessment

To assess the level of functional disability, we adopted the two most common scales used in patients with ABI: the LCF and the DRS.

The LCF assesses the patients’ cognitive–behavioural profile, ranging from 1 (No Response) to 8 (“Purposeful/Appropriate Response”). Importantly, an LCF score ≥ 7 (index of “Automatic/Appropriate Response”) has been used as one of the inclusion criteria.

The DRS was used to describe the patients’ level of functional disability. It evaluates disability at three levels: Impairment, captured by the first three items (“Eye Opening,” “Communication Ability” and “Motor Response”) is a slight modification of the GCS. Disability is captured by questions evaluating cognitive ability for “Feeding,” “Toileting” and “Grooming”. The final level, Handicap, captures “Level of Functioning”, using a modified measure developed by [39], and “Employability” [38].

DRS total scores allow for patient classification into the following disability categories: 0 = None; 1 = Mild; 2–3 = Partial; 4–6 = Moderate; 7–11 = Moderately Severe; 12–16 = Severe; 17–21= Extremely Severe; 22–24 = Vegetative State; 25–29 = Extreme Vegetative State. Higher DRS scores correspond to higher levels of disability [38].

#### 2.3.2. Self-Awareness Assessment

The Patient Competency Rating Scale (PCRS) was used to assess the level of metacognitive SA [19] because of its psychometric properties and feasibility [40,41]. The PCRS was translated and validated in Italian in the past by Ciurli et al. (2010) [42]. The PCRS is a 30-item self-report questionnaire that requires patients and their relatives to make an independent judgment of perceived degree of competency demonstrated in several behavioural, cognitive and emotional situations using a 5-point Likert scale (ranging from 1, “Can’t do”, to 5, “Can do with ease”). Total PCRS scores range from 30 to 150, with higher scores indicating higher levels of perceived competency. Thus, the level of patients’ metacognitive SA (i.e., the PCRS discrepancy score—PCRS^DS^) is obtained by subtracting the patient’s PCRS total score from the relative’s PCRS total score [17,43].

To compare inpatients and DH patients on the same abilities, we administered the PCRS-NR, a briefer version of the PCRS used in inpatient neurorehabilitation unit [36]. The PCRS-NR excludes all items regarding patients’ competencies in activities of daily living outside the hospital. Thus, the PCRS-NR assesses metacognitive SA across three domains: cognitive (3 items), interpersonal (5 items) and emotional (5 items). Total PCRS-NR scores can range from 13 to 65.

In line with previous findings [18,37], we adopted a PCRS-NR^DS^ cut-off score of 5 points (scores ≥ 5 indicating low metacognitive SA) to differentiate between high-metacognitive SA and low-metacognitive-SA patients.

#### 2.3.3. Psychological and Neuropsychiatric Assessment

##### Anosodiaphoria

To assess both ISA and anosodiaphoria with the same measurement scale over time, Bivona et al. [18] created an extended Italian version of the PCRS-NR, that is, the “Anosodiaphoria Extension of the PCRS-NR” (AE-PCRS-NR). Anosodiaphoria is related to the patients’ subjective emotional state regarding the level of their competencies (impaired or reduced competencies).

For each of the PCRS items, patients were also asked to complete the anosodiaphoria section, which explores their level of concern about their perceived level of performance on a Likert scale ranging from 5 (“I am not disturbed/worried at all”) to 1 (“I am very disturbed/worried”). For example, after completing PCRS-NR “item 1” (“How much of a problem do I have in preparing my own meals?”), patients were also asked to answer the question (“How much am I disturbed/worried about my ability to prepare my own meals?”). The total AE-PCRS-NR score can range from 13 to 65, with higher scores indicating higher levels of anosodiaphoria [18].

To obtain an objective measure of anosodiaphoria concerning the actual level of patients’ difficulties, Bivona et al. [18] computed an Anosodiaphoria Index (AE-PCRS Index) by dividing the patients’ AE-PCRS-NR total score by the relatives’ PCRS-NR total score, considering the latter as an external/“objective” measure of patients’ difficulties. An AE-PCRS-NR Index > 1 indicates the presence of anosodiaphoria, with a maximum AE-Index score of 5 obtained in the case where a patient reports the maximum score on the AE-PCRS-NR (i.e., =65, representing total lack of concern) and their relatives report a minimum total score on the PCRS-NR (i.e., =13, representing the highest level of functional difficulties).

##### Apathy

To assess apathy, we administered the Italian version of the Apathy Evaluation Scale (AES) [44]. The AES [45] is an 18-item scale that assesses the behavioural, emotional, and cognitive dimensions of apathy. Each item is rated on a 4-point Likert scale ranging from 0 (“not at all true”) to 4 (“very true”). This scale can be administered in a self-report version (AES-S), by relatives or caregivers (AES-I), or by a clinician following a semi-structured interview with the patients (AES-C). In the present study, we used the AES-C version (“AES” from now on). Lane-Brown and Tate, (2009), using a sample of patients with TBI, suggested that an AES score ≥ 37 indicates the presence of apathy [46].

##### Anxiety

We administered the State-Trait Anxiety Inventory (STAI, X1-X2) [47] to assess self-reported anxiety. The STAI is a self-report scale that measures two distinct components of anxiety: the current state of anxiety (STAI-X1), and anxiety as a trait (STAI-X2). STAI-X1 consists of 20 descriptive statements regarding how the participant feels currently, including the moment of the interview. STAI-X2 consists of 20 descriptive statements regarding how the participant usually feels. For both sub-scales, the total score can range from 20 (very low anxiety) to 80 (very high anxiety). A score = 40 for each subscale is considered the cut-off for a pathological level of anxiety.

##### Depression

The Beck Depression Inventory (BDI) [48] includes 21 items that assess self-reported cognitive, behavioural, affective, and somatic components of depression. Higher scores represent higher levels of reported depression. The BDI is one of the most widely used measures of depression. It has been shown to effectively differentiate between non-depressed, moderately depressed, and severely depressed adults [48] and it demonstrates good psychometric properties [49,50]. The standard cut-off scores proposed by Beck et al. [51] are as follows: 0–9 indicates minimal depression; 10–16 indicates mild depression; 17–29 indicates moderate depression; 30–63 indicates severe depression.

##### Post-Traumatic Stress Disorders Symptoms

To evaluate the traumatic impact of the neurological event qualifying patients for this study, based on DSM-5 criteria for PTSD, we administered the Impact of the Event Scale-revised (IES-R) [52]. The IES-R is a self-report measure of current subjective distress, that assesses the intensity of emotional and cognitive reactions in response to a specific traumatic event. It comprises three subscales representative of the major symptom clusters of PTSD: intrusion, avoidance, and hyper-arousal. A score > 24 is an index of the presence of PTSD symptoms.

### 2.4. Statistics

The different sample size of each patient group, visual inspection of histograms, and the Kolmogorov–Smirnov test of normality for emotional scores revealed the groups were not normally distributed (*p* < 0.01). Accordingly, to investigate possible between-groups differences on psychometric scales, first the Kruskal–Wallis H test was used to compare the three patient groups for each psycho-emotional score. When a significant effect was found, pairwise comparisons were performed using Mann–Whitney U tests.

Effect sizes (Rosenthal’s r) were calculated for the pairwise comparisons. We considered Cohen’s classification of the effect sizes as small (r = 0.1), medium (r = 0.3), and large (r ≥ 0.5) [53]. Finally, the sociodemographic variables and the psycho-emotional scores were submitted to bivariate correlation analysis. Spearman’s rho coefficients were computed.

## 3. Results

Table 1 shows the means, standard deviations, medians, and minimum and maximum values for each sociodemographic and emotional variable in the three groups.

### 3.1. Sociodemographic Variables and Chronicity

A Kruskal–Wallis H test was conducted to examine group differences in each sociodemographic variable among the three patient groups. Statistically significant differences emerged for age [χ^2^(2) = 6.544, *p* = 0.038] and education [χ^2^(2) = 7.935, *p* = 0.019]. As for chronicity, no significant difference [χ^2^(2) = 0.137, *p* = 0.934] was found.

As shown in Table 2, patients in Group 1 were significantly older than those in Groups 2 (Z = −2.413, *p* < 0.01; medium effect size) and 3 (Z = −1.776, *p* < 0.05; medium effect size).

In terms of education level, Group 1 and 2 were significantly different (Z = −2.456, *p* < 0.01; medium effect size) as were Group 2 and 3 (Z = −2.092, *p* < 0.05; medium effect size).

### 3.2. Differences Between-Groups on Psycho-Emotional Variables

Kruskal–Wallis H test was conducted to examine group differences in each variable among the three patient groups. As for apathy, no significant differences were found [χ^2^(2) = 2.081, *p* = 0.353].

Conversely, statistically significant differences emerged for anosodiaphoria [χ^2^(2) = 25.986, *p* < 0.001], state anxiety [χ^2^(2) = 9.444, *p* = 0.009] and trait anxiety [χ^2^(2) = 9.006, *p* = 0.011] (STAI-X1 and STAI-X2, respectively), depression [χ^2^(2) = 7.324, *p* = 0.026], and PTSD symptoms [χ^2^(2) = 12.176, *p* = 0.002].

Consecutively, when a significant effect was found, pairwise comparisons were performed using Mann–Whitney U tests. As shown in Table 3, for all relationships other than those involving AES (i.e., apathy), results from the Mann–Whitney U test showed significant differences in the psycho-emotional scales between Groups 1 (i.e., patients with low metacognitive SA) and 3 (i.e., patients with none-to-moderate disability), and between Groups 1 and 2 (i.e., patients with moderately severe-to-severe disability).

In particular, Group 1 exhibited higher anosodiaphoria (Z = −4.778, *p* < 0.001; large effect size), and lower state (Z = −1.653, *p* < 0.05; small effect size) and trait anxiety (Z = −2.636, *p* < 0.01; medium effect size), as well as lower depression scores (Z = −1.690, *p* < 0.05; small effect size) than Group 3.

The same pattern emerged from the comparison between Groups 1 and 2: patients in Group 1 showed significantly higher anosodiaphoria (Z = −3.929, *p* < 0.001), and lower state (Z = −2.798, *p* < 0.01) and trait (Z = −2.760, *p* < 0.01) anxiety and depression scores (Z = −2.764, *p* < 0.01) than those in Group 2. The effect size was almost large in all cases.

Finally, the comparison between Groups 2 and 3 showed significant differences only in the state anxiety STAI-X1 (Z = −1.952, *p* < 0.05) and in the IES-R scores (Z = −2.287, *p* < 0.05), indicating higher state anxiety and more PTSD symptoms, respectively, in Group 2. In both cases, the effect size was medium.

### 3.3. Correlation Analysis Result

The correlational analysis performed to exclude any possible influence of sociodemographic variables on the differences between groups found on the psycho-emotional features between groups showed that age correlated positively only with the PCRS-NR^DS^ (ρ = 0.42; *p* < 0.001)—specifically, the higher the age, the lower the metacognitive SA. No correlations emerged between any sociodemographic variables and depression, state and trait anxiety, anosodiaphoria, apathy, and PTSD symptoms (see Table 4).

## 4. Discussion

The present study investigated the relationship between level of impaired self-awareness and emotional states in patients with moderate-to-severe ABI.

We also investigated the association between the level of functional disability and anxiety and depression and PTSD symptoms in high-metacognitive-SA patients. To achieve this, participants were divided into low metacognitive SA or high metacognitive SA based on a PCRS-NR^DS^ cut-off consistent with prior literature [18,37]. We further divided participants with high metacognitive SA according to their level of functional disability, as classified by the DRS, into patients with none-to-moderate disability and moderately severe-to-severe disability. We named these three sub-groups Group 1, Group 2, and Group 3, respectively.

In line with previous studies, high-metacognitive-SA patients showed higher STAI scores—indicating higher level of anxiety [17,24,25,26], and higher BDI scores—indicating higher levels of depressive symptoms [17,21,22,23,24,32,54]. We predicted these findings believing that only patients who are aware of one or more functional (e.g., cognitive, physical, etc.) difficulties would present with anxiety and depression as congruent reactions to the event. Indeed, as stated by Prigatano et al. [55], these disorders should be considered in the patients’ adaptive reactions to the injury that, if adequately treated, can lead to better adjustment to its consequences over time, and decreased frustration, confusion, and distress provoked by the ABI. Additionally, low-metacognitive-SA patients showed significant higher anosodiaphoria scores than those in Groups 2 and 3 (i.e., high-metacognitive-SA patients). Finally, no differences in terms of apathy scores emerged between the three groups.

The presence of significantly higher anosodiaphoria (but not of apathy) scores in low-metacognitive-SA patients, compared to high-metacognitive-SA patients, aligns with the results of a recent studies with similar findings [18]. This may suggest that, although similar to each other, apathy and anosodiaphoria may be related to overlapping (e.g., the insula), but also different brain regions [54,56]. We predicted the association between low-metacognitive SA and anosodiaphoria since observing emotional concern in patients who are not self-aware of their difficulty can be considered nonsense.

Of note, some patients with good metacognitive SA also presented with anosodiaphoria (namely, some AE-PCRS-NR Index scores in the high-metacognitive-SA patients groups were above the cut-off). Indeed, as shown in Table 1, the maximum scores in Groups 2 and 3 were 1.24 and 1.39, respectively. This finding aligns with our clinical practice, since we find anosodiaphoria as a negative reaction to the ABI even in patients with good metacognitive SA and, as such, it represents another relevant obstacle in neurorehabilitation.

We did not find significant differences in levels of anxiety and depression between groups, in relation to the level of functional impairment as measured by the DRS, other than for *state* anxiety. This finding suggests that even low difficulties may be emotionally distressing, likely due to a patient’s fear of not recovering their complete level of pre-ABI functioning. Other studies [31,32] found divergent results, since higher levels of self-reported anxiety and depression were associated only with higher levels of functional impairment. These different results could be based on the different sample enrolled in terms of ABI severity in the acute phase (i.e., they included also patients with mild ABI), or to the measures used to assess the level of functional disability (i.e., they used subjective assessment methods of post-ABI consequences, rather than an objective measure such as the DRS, that we used in the present study).

As for the significant difference between the two groups with high metacognitive SA in terms of *state* anxiety (i.e., the higher the disability, the higher the state anxiety), it should be considered that patients with moderately severe-to-severe disability were young adults (with a median age of 45 years) and, as such, with a long life expectancy. Therefore, the higher *state* anxiety found in patients with higher DRS scores could be related to items from the assessment (e.g., dependence on others, psychosocial adaptability in terms of the possibility to return to work). Consensus on the association between anxiety and return to work is still a matter of debate [57,58], and further studies are needed.

With regard to the subjective impact of the ABI in terms of PTSD symptoms, we chose to focus only on high-metacognitive-SA patients. This was decided because ISA is a potentially confounding variable, which can make diagnosing PTSD difficult [33]. Also, since we used a self-report tool to explore PTSD symptoms, investigating this disorder in an individual with ISA may be paradoxical [8], increasing the risk of misdiagnosis [33,59]. Thus, we explored the potential presence of PTSD symptoms only in high-metacognitive-SA patients, by comparing Groups 2 and 3. We found that higher levels of functional disability were significantly associated with higher presence of PTSD symptoms (i.e., patients with moderately severe-to-severe disability lived more dramatically the brain injury experience). This aligns with our prediction, since being self-aware of higher difficulties associated with the brain injury may increase the risk of developing PTSD symptoms (i.e., intrusiveness, avoidance, hyper-arousal) as measured by the IES-R scale. In fact, at least three of the DSM-5 PTSD diagnostic criteria are compatible with severe ABI. Indeed, the brain injury can be considered “an event that is threatening to one’s wellbeing” [criterion a]. Moreover, some post-severe TBI symptoms (such as insomnia, irritability, concentration difficulties, hypervigilance, or heightened startle response) are also typical persisting signs of marked arousal, which may cause marked impairment to one’s functioning [criteria d and e] [60]. Although symptoms of PTSD have already been found in patients with moderate-to-severe ABI [33,61,62,63], to the best of our knowledge, no previous studies investigated the possible presence of PTSD symptoms according to the level of functional disability, which is an innovative contribution of the present study.

Finally, since we found some significant socio-demographic differences between-groups (i.e., in terms of age between Group 1 and both Groups 2 and 3, and in terms of educational level between Groups 1 and 3, and between Groups 2 and 3), we performed a correlational analysis to exclude any possible influence of these socio-demographic variables on the psycho-emotional feature. The analysis revealed that only age correlated with PCRS-NR^DS^ (namely, with lower levels of metacognitive SA), whereas no other significant relationships were found.

The present study presents some important limits. First, the sample size of each sub-group was low. Furthermore, 41 out of 60 patients were inpatients, while 19 were treated in DH regimen; thus, it is possible that inpatients were more likely to have lower SA, determining as a consequence an evaluation bias. Accordingly, our findings should be considered as preliminary; further studies with larger sub-samples are needed to confirm and generalize our results.

Second, we did not provide neuroimaging data that could account for the absence of apathy in the presence of anosodiaphoria symptoms found in our study (e.g., different brain lesions sites).

Thirdly, according to the above-mentioned classificatory models [14,15], we considered only the metacognitive level of SA through the PCRS-NR discrepancy scores between patients and caregivers. Consequently, it must be considered that the subjective opinions and expectations of caregivers may also have an influence, leading (at least in part) to possible misdiagnosis. Other studies should also consider the relationship between the psycho-emotional variables here investigated and emergent/online SA, as well as the other levels of SA (i.e., *declarative* and *real anticipatory* SA) according to a partially new classificatory model proposed by Bivona and Villalobos [19].

Importantly, ISA is one of the main problems not only in the field of ABI, but also in other clinical populations (e.g., neurodegenerative [64] and psychiatrics [65,66,67]). Given the small samples size, further studies are needed to confirm our findings, through rigorous process observations and longitudinal research.

However, despite these limits, we believe that some important cues arose from our work. Our study highlighted relevant differences between groups according to the levels of metacognitive SA, in terms of anxiety and depression (significantly higher in high-metacognitive-SA patients) and anosodiaphoria (significantly higher in low-metacognitive-SA patients).

Moreover, in line with previous findings by Bivona and colleagues [17], our study confirmed a dissociation between apathy and anosodiaphoria in ISA patients, which suggests the need for an accurate differential diagnosis to implement an optimal neurorehabilitation project.

Finally, in high-metacognitive-SA patients, a complete neuropsychological and neuropsychiatric assessment should pay particular attention to the possible presence of PTSD symptoms, especially in patients with higher functional disability.

## 5. Conclusions

Although preliminary, the present study confirmed the importance of diagnosing, as early and specifically as possible, both levels of ISA and dysfunctional emotional reactions, such as anosodiaphoria, in patients with moderate-to-severe ABI.

We found adaptive emotional reactions (i.e., anxiety and depression symptoms) in high-metacognitive-SA patients, regardless of the level of functional disability, as well as PTSD symptoms associated with higher functional disability.

Accordingly, although the mechanisms by which SA interventions exert their effects are still a matter of debate (for details, see a recent systematic review and meta-analysis by Villalobos et al. [68]), our findings confirm the importance of first treating ISA and anosodiaphoria. Second, when patients show adequate levels of SA, the rehabilitation project should address not only the cognitive impairment, but also the psycho-emotional distress, such as anxiety and depression and the possible presence of PTSD symptoms.

In this vein, psychological support plays a crucial role in neurorehabilitation hospitals, helping to address psychological challenges closely linked to the recovery process, potentially involving not only the patients but also their families.

## Figures and Tables

**Table 1:** Means, standard deviations, medians, minimum and maximum values for each sociodemographic and emotional variable in the three groups.

	Group 1 (N = 17)					Group 2 (N = 15)					Group 3 (N = 28)				
	Mean	SD	Median	Min	Max	Mean	SD	Median	Min	Max	Mean	SD	Median	Min	Max
Age (years)	50.65	12.33	55	26	63	42.07	15.7	45	21	65	38.04	16.46	33.5	18	65
Education (years)	14.35	2.78	13	8	19	13.8	2.3	13	8	17	12.04	2.91	13	8	17
Chronicity (days)	168.18	166.32	105	37	698	174	196.7	79	26	772	167.04	147.94	87	33	489
AE-PCRS-NR Index	1.29	0.16	1.27	1.1	1.71	1.03	0.14	1.02	0.8	1.24	0.98	0.18	0.99	0.5	1.39
STAI-X1	32.12	9.78	31	21	55	44.47	13.83	43	25	79	36.61	10.88	33.5	21	63
STAI-X2	32.35	9.12	30	21	59	44.73	12.15	43	26	62	40.93	11.52	41.5	20	66
BDI	6.59	6.27	5	0	25	12.60	7.48	12	3	33	10.29	8.02	9.5	0	29
AES	30.0	7.73	29	18	46	29.07	8.64	29	20	52	31.78	7.75	31	18	47
IES-R	--	--	--	--	--	26.80	15.11	27	5	58	16.74	14.60	14	1	69

Note. SD: standard deviation; Group 1: *low*-metacognitive-SA patients. Group 2: *high*-metacognitive-SA patients and *moderately severe-to-severe* disability. Group 3: *high*-metacognitive-SA patients and *none-to-moderate* disability. AE-PCRS-NR Index: Anosodiaphoria Extension of the Patient Competency Rating Scale-Neurorehabilitation form—Index. STAI-X1: State-Trait Anxiety Inventory—*State* anxiety. STAI-X2: State-Trait Anxiety Inventory—*Trait* anxiety. BDI: Beck Depression Inventory. AES: Apathy Evaluation Scale. IES-R: Impact of the Event Scale-Revised.

**Table 2:** Comparison between groups on sociodemographic variables and chronicity (Mann–Whitney U test, *p* value and effect size are reported).

	Group 1 vs. Group 2			Group 1 vs. Group 3			Group 2 vs. Group 3		
	Z	*p* Value	ES	Z	*p* Value	ES	Z	*p* Value	ES
Age	−1.776	0.038 *	0.314	−2.413	0.008 **	0.359	−0.829	0.203	0.126
Education	−0.473	0.341	0.084	−2.456	0.007 **	0.366	−2.092	0.018 *	0.319
Chronicity	−0.359	0.368	0.063	−0.082	0.467	0.012	−0.293	0.384	0.045

Note. Group 1: *low*-metacognitive-SA patients. Group 2: *high*-metacognitive-SA patients and *moderately severe-to-severe disability.* Group 3: *high*-metacognitive-SA patients and *none-to-moderate disability*. ES: effect size (Rosenthal’s r). * *p* < 0.05; ** *p* < 0.01.

**Table 3:** Comparison between groups on psycho-emotional variables (Mann–Whitney U test, *p* value and effect size are reported).

	Group 1 vs. Group 2			Group 1 vs. Group 3			Group 2 vs. Group 3		
	Z	*p* Value	ES	Z	*p* Value	ES	Z	*p* Value	ES
AE-PCRS-NR Index	−3.929	<0.001 **	0.694	−4.778	<0.001 **	0.712	−0.829	0.203	0.126
STAI-X1	−2.798	0.003 **	0.495	−1.653	0.049 *	0.246	−1.952	0.025 *	0.298
STAI-X2	−2.760	0.003 **	0.488	−2.636	0.004 **	0.393	−0.905	0.182	0.138
BDI	−2.764	0.003 **	0.489	−1.690	0.045 *	0.252	−1.212	0.112	0.185
AES	−0.516	0.303	0.091	−0.801	0.211	0.119	−1.437	0.075	0.219
IES-R							−2.287	0.011 *	0.349

Note. Group 1: *low*-metacognitive-SA patients. Group 2: patients with *high* metacognitive SA and *moderately severe-to-severe disability*. Group 3: patients with *high* metacognitive SA and *none-to-moderate disability*. ES: effect size (Rosenthal’s r). AE-PCRS-NR Index: Anosodiaphoria Extension of the Patient Competency Rating Scale-Neurorehabilitation form—Index. STAI-X1: State-Trait Anxiety Inventory—*State* anxiety. STAI-X2: State-Trait Anxiety Inventory—*Trait* anxiety. BDI: Beck Depression Inventory. AES: Apathy Evaluation Scale. IES-R: Impact of the Event Scale-Revised; * *p* < 0.05; ** *p* < 0.01.

**Table 4:** Rho Spearman bivariate correlation between sociodemographic and psycho-emotional variables.

	PCRS-NR^DS^	AE-PCRS Index	STAI-X1	STAI-X2	BDI	AES	IES-R
Age	0.422 **	0.248	0.076	−0.121	−0.02	−0.246	−0.081
Education	0.113	0.087	0.145	0.009	0.072	−0.16	0.092
Chronicity	−0.021	0.194	0.052	0.152	0.099	0.184	0.254

Note: PCRS-NR^DS^: Patient Competency Rating Scale-Neurorehabilitation Form Discrepancy Score. AE-PCRS-NR Index: Anosodiaphoria Extension of the Patient Competency Rating Scale-Neurorehabilitation form—Index. STAI-X1: State-Trait Anxiety Inventory—*State* anxiety. STAI-X2: State-Trait Anxiety Inventory—*Trait* anxiety. BDI: Beck Depression Inventory. AES: Apathy Evaluation Scale. IES-R: Impact of the Event Scale-Revised; ** *p* < 0.01.

## Data Availability

The data presented in this study are available on request from the corresponding author. Data are unavailable due to privacy or ethical restrictions.
